# Impacts of Myrtle Rust Induced Tree Mortality on Species and Functional Richness within Seedling Communities of a Wet Sclerophyll Forest in Eastern Australia

**DOI:** 10.3390/plants12101970

**Published:** 2023-05-12

**Authors:** Kristy Stevenson, Geoff Pegg, Jarrah Wills, John Herbohn, Jennifer Firn

**Affiliations:** 1 School of Agriculture and Food Science, The University of Queensland, St Lucia, QLD 4072, Australia; 2 The Queensland Department of Agriculture and Fisheries, Brisbane, QLD 4001, Australia; 3 Australian Government Department of Climate Change, Energy, the Environment and Water, Canberra, ACT 2601, Australia; 4 Tropical Forests and People Research Centre, University of the Sunshine Coast, 90 Sippy Downs Drive, Sippy Downs, QLD 4556, Australia; 5 School of Biological and Environmental Science, Queensland University of Technology, Brisbane, QLD 4001, Australia; jennifer.firn@qut.edu.au

**Keywords:** *Austropuccinia psidii*, disturbance, species richness

## Abstract

*Austropuccinia psidii* is an introduced plant pathogen known to have caused significant declines in populations of several Australian native Myrtaceae species. However, limited research has focused on the impacts of the pathogen on plant communities in the aftermath of its invasion. This study investigated the relationship between disease impact level, plant species diversity, and functional richness in seedling communities in a wet sclerophyll forest in southeast Queensland. A clear shift was found from early colonizer Myrtaceae species in the mid- and understory to a more diverse non-Myrtaceae seedling community indicative of secondary succession. Comparisons of key Myrtaceae species and the seedling community suggest that there may also be a shift towards species that produce drupes and larger seeds, and overall, a current reduction in fruit availability due to the dramatic loss of previously dominant species. Seedling diversity showed no significant correlation with tree mortality, possibly due to favorable rainfall conditions during the study period. The more subtle changes in forest composition, such as changes in fruit type and availability due to myrtle rust, however, could affect the visitation of local bird species in the short term and certainly reduce the store of early colonizing native shrub and tree species.

## 1. Introduction

**Figure 1:**
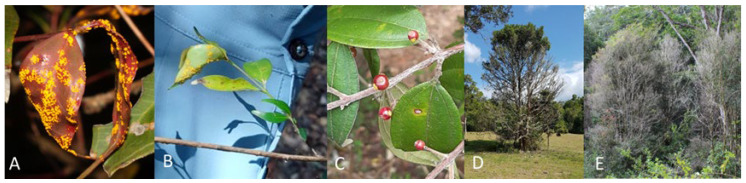
Photos of *Austropuccinia psidii* infection and impacts at the study site. (**A**) Infection on leaves of *Rhodamnia maideniana* C.T.White. (**B**) Infection on leaves of *Gossia hillii* (Benth.) N. Snow & Guymer. (**C**) Infection on fruits of *Rhodamnia rubescens*. (**D**) Branch dieback due to repeated *Austropuccinia psidii* infection on *Syzygium corynanthum* (F.Muell.) L.A.S. Johnson. (**E**) Trees killed by repeated *Austropuccinia psidii* infection.

Invasive plant pathogens can cause significant changes in their host populations. Global trade and travel facilitate the movement of plant pathogens into new habitats [1,2]. Some of these pathogens establish and cause high mortality in local host populations. For example, *Cryphonectria parasitica*, the causal agent of chestnut blight, decimated populations of *Castanea dentata* (Marshall) Borkh after its establishment in forests in the United States [3]. Similarly, *Phytophthora ramorum* has caused the deaths of an estimated 17.5 million *Notholithocarpus densiflorus (Hook. & Arn.) Manos*, *Cannon, & S.H. Oh* in California and Oregon [4]. In Australia, *Austropuccinia psidii*, the causal agent of myrtle rust, has had a similar impact. Since being detected in Australia in 2010, myrtle rust has caused significant population declines in several tree and shrub species, including *Rhodomyrtus psidioides* Benth. and *Rhodamnia rubescens* (Benth.) Miq. [5,6]. Myrtle rust affects the new leaves and stems of tree hosts, as well as the flowers and fruits of some species (Figure 1A–C). Repeated infection can result in branch dieback and eventual tree death in highly susceptible individuals (Figure 1D–E). Research on the impact of myrtle rust on the composition of plant communities is limited to a few studies, with most focused on the change in abundance and composition of Myrtaceae species [7]. Further research is needed to understand myrtle rust impacts on the resultant plant community and the overall resilience of forest ecosystems, with a particular focus on the seedling layer, as individuals from this community may eventually establish and replace the trees killed by myrtle rust.

Species richness is a key characteristic of forest communities; however, evidence of the impact of introduced pathogens on species richness remains equivocal. The identity and relative abundance of plant species that make up a forest community can impact ecosystem functioning [8]. A general positive relationship between the quantity of biodiversity and several forest functions, including productivity and the provision of habitat and resources for fauna, has been reported [9,10]. Consequently, investigating how invasive species, such as introduced pathogens, affect plant species composition is important. There is evidence from several ecosystems that introduced plant pathogens can modify the overall species composition of native forests [11,12]. For example, the establishment of *Phytophthora cinnamomi* in some Australian forests has induced positive [13], negative [14], and neutral effects [13,15] on species richness. While there is strong evidence that myrtle rust is affecting the abundance of Myrtaceae species, for example, critically endangering populations of species such as *Rhodomyrtus psidioides* [6], there is limited information available on how these declines are affecting species richness at the plant community level. Fernandez-Winzer et al. [16] investigated the impacts of myrtle rust on plant species richness in Australia, with a focus on rainforest communities. A lower species richness and seedling abundance were recorded in plots with *Rhodamnia rubescens* canopies infected by myrtle rust when compared to plots with healthy canopies. Further research is needed to determine the impacts of myrtle rust on species richness in a wider range of forest communities in Australia.

The potential loss or even gain of plant species in a forest due to pathogen infections is important because different plant species bring different prospects for growth, survival, and reproductive traits to forests. The richness of plant functional traits within a community therefore influences key ecosystem functions and services. Measured at the individual plant level, a plant trait is an attribute, such as seed size, that influences plant growth, survival, and reproduction and, in turn, can indirectly influence plant fitness [17]. While functional diversity can indicate the richness of trait values or trait states within a plant community for a particular function [18]. For example, a community where all plant species express the same trait state for dispersal (e.g., wind) would have a lower functional richness than a community where species express different types of dispersal traits (e.g., wind, bird, water).

The richness of plant functional traits within a community can provide some insight into the functioning of the ecosystem and the ecosystem services it provides [10,19]. For example, reproductive and dispersal traits, such as seed size, fruit size, or dispersal mechanism, are key to understanding cross-trophic interactions, such as plant-disperser interactions [20,21]. The richness of functional traits can change in response to disturbance and land-use change, initiating a shift in the strength and importance of environmental filters and competition [22]. For example, disturbances that reduce canopy layers in a forest, such as that caused by myrtle rust, might lead to a decrease in functional trait richness because environmental conditions such as light become more homogenous and filter for particular traits.

There is limited research on the impacts of introduced pathogens on the richness of plant functional traits. There is growing evidence that invasive plant pathogens can affect the very functions and food webs that make up ecosystems, particularly cross-trophic-level interactions. For example, changes in the richness or composition of bird guilds have been reported in response to the establishment of *Phytophthora cinnamomi* in Australian forests and in Hawaiian forests after the establishment of the Rapid Ohia Death [23,24]. No such study has been conducted in myrtle rust-infected forests. Similarly, no study has investigated how the richness of dispersal and reproductive traits, those linked to cross-trophic-level interactions, are affected by myrtle rust. Research investigating the possible effects of the pathogen on these traits will aid in determining how the pathogen is shaping forest communities and impacting local fauna.

This study adds to existing research on the impacts of myrtle rust on seedling communities undertaken by Fernandez-Winzer et al. [16] in Australian rainforests with the selection of a site that supports a different forest type, a wet sclerophyll forest. The forest at the study site has an overstory of *Eucalyptus* sp. and other functionally similar genera that appear tolerant to myrtle rust and a mid-and understory dominated by rainforest species from the Myrtaceae family that have a high susceptibility to myrtle rust infection. This study aims to investigate three key questions: (1) Is there a correlation between disease impact level and species richness in seedling communities? (2) Is there a correlation between disease impact level and functional richness in seedling communities? (3) Is the decline of susceptible Myrtaceae species likely to cause a change in dominance of key plant traits, e.g., bird dispersal to wind dispersal?

## 2. Results

**Figure 2:**
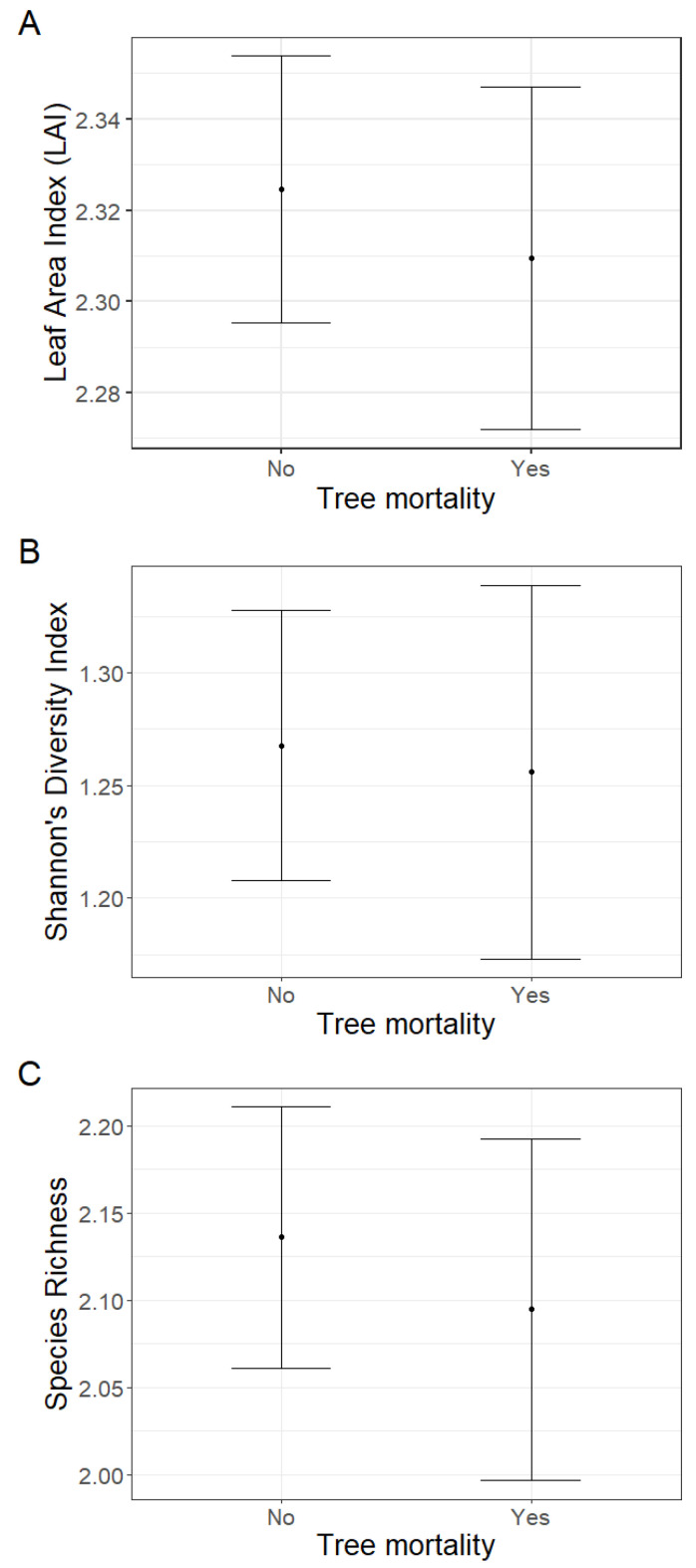
There were negligible effects of tree mortality on (**A**) Leaf Area Index (LAI), (**B**) Shannon’s diversity index, or (**C**) species richness when comparing the mean fixed effects and standard error estimates.

**Figure 3:**
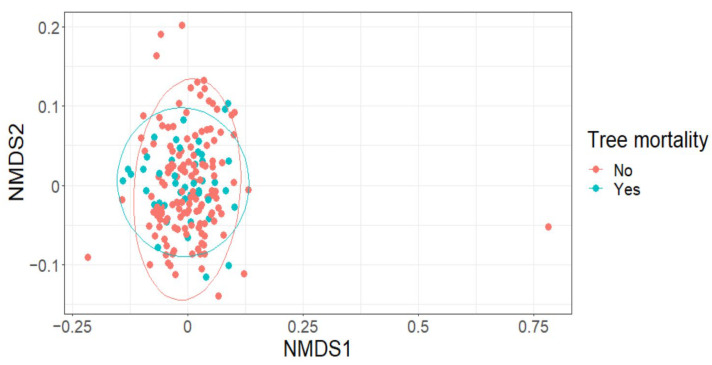
Tree mortality (yes/no) was not observed to markedly affect the grouping of plots using species composition data.

The effect of tree mortality was negligible on Leaf Area Index (LAI) (fixed effect = −0.02, standard error = 0.03, t = −0.46; Figure 2A), Shannon’s diversity index (fixed effect = −0.01, standard error = 0.08, t = −0.15; Figure 2B) or species richness (fixed effect = −0.04, standard error = 0.09, t = −0.47) (Figure 2C). Further, tree mortality did not appear to have a marked effect on the grouping of plots based on species composition using nMDS analysis (k = 2, stress = 0.22; Figure 3), nor was it observed to have a significant effect using a PerMANOVA analysis (F = 1.60, *p =* 0.14).

**Figure 4:**
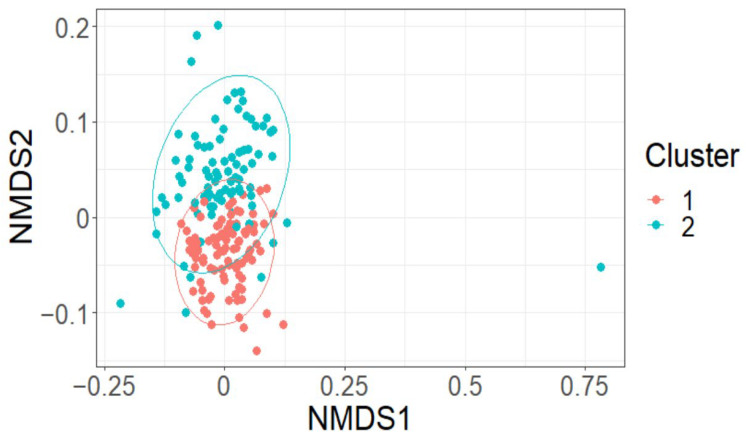
Moderate grouping of plots was observed when overlying cluster groups on nMDS created using species composition data.

**Figure A1:**
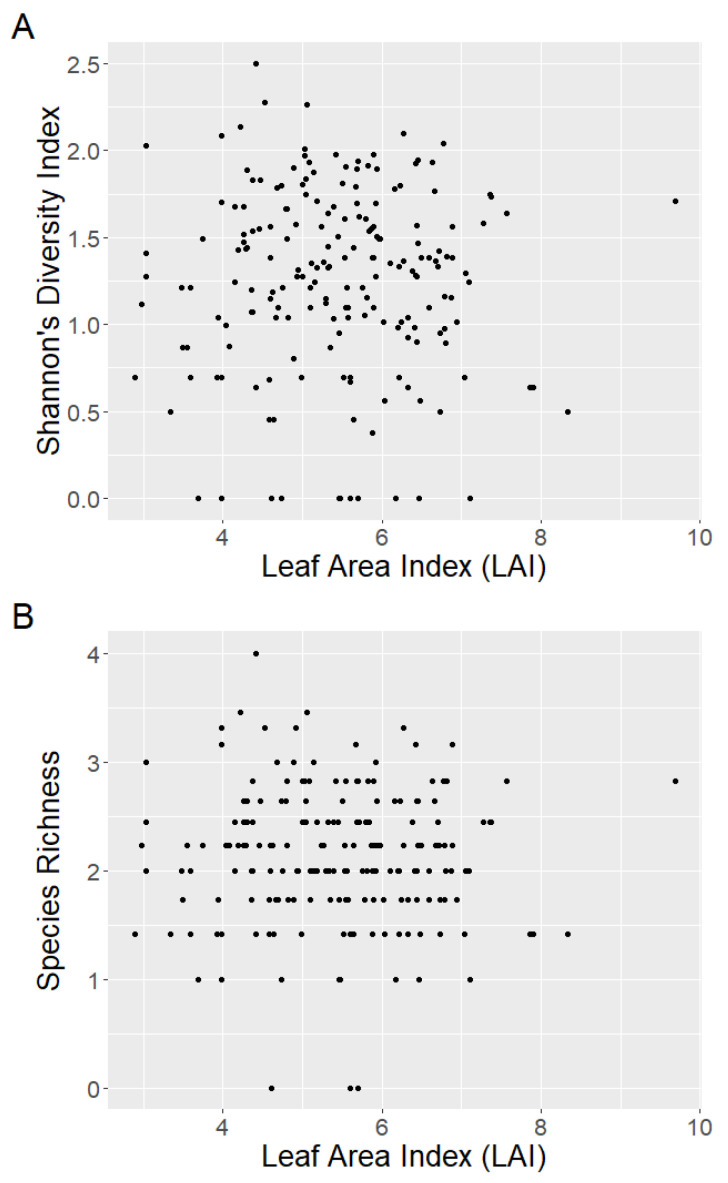
There were negligible effects of the Leaf Area Index (LAI) on (**A**) Shannon’s diversity index and (**B**) species richness when comparing the mean fixed effects and standard error estimates.

**Figure A2:**
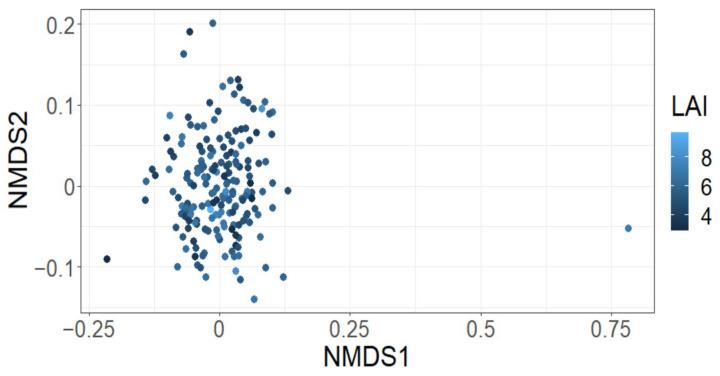
No distinct grouping of plots (k = 2, stress = 0.22) in response to LAI was observed.

The effect of LAI was negligible on both Shannon’s diversity index (fixed effect = −0.03, standard error = 0.04, t = −0.88) and on species richness (fixed effect = −0.06, standard error = 0.04, t = −1.37) (Appendix A, Figure A1). This was supported by multivariate analysis. LAI did not appear to markedly affect the grouping of plots based on species composition using nMDS analysis (k = 2, stress = 0.22; Appendix B, Figure A2). Two clusters of plots based on species composition were determined using cluster analysis. A significant difference between the two clusters (F = 23.61, *p* < 0.01) was observed using perMANOVA analysis and highlighted when groups were overlaid on the nMDS plot (Figure 4). This clustering was driven by a difference in species richness, as highlighted by a significant effect of group on species richness (F = 8.92, *p* < 0.01) and Shannon’s richness index (F = 11.64, *p* < 0.01) using linear mixed-effects models. Indicator species analysis suggested that several species were significant drivers of the clustering. The key driver of group 1 was *Acmena smithii* (Poir.) Merr. & L.M. Perry (InVal = 0.67, *p* < 0.01). While key drivers of group 2 were *Syzygium oleosum* (F. Muell.) B.Hyland (InVal = 0.63, *p* < 0.01), *Glochidion ferdinandi* (Müll.Arg.) F.M. Bailey (InVal = 0.62, *p* < 0.01), *Elaeocarpus obovatus* G. Don (InVal = 0.43, *p* < 0.01), *Cryptocarya microneura* Meisn. (InVal = 0.40, *p* < 0.01), *Acacia disparrima* M.W. McDonald & Maslin (InVal = 0.32, *p* < 0.01), *Gossia hillii* (InVal = 0.29, *p =* 0.01), *Toona ciliata* M. Roem. (InVal = 0.28, *p =* 0.04), *Wilkiea huegeliana* (Tul.) A.DC. (InVal = 0.27, *p =* 0.03) and *Cryptocarya laevigata* Blume (InVal = 0.26, *p =* 0.01).

**Figure 5:**
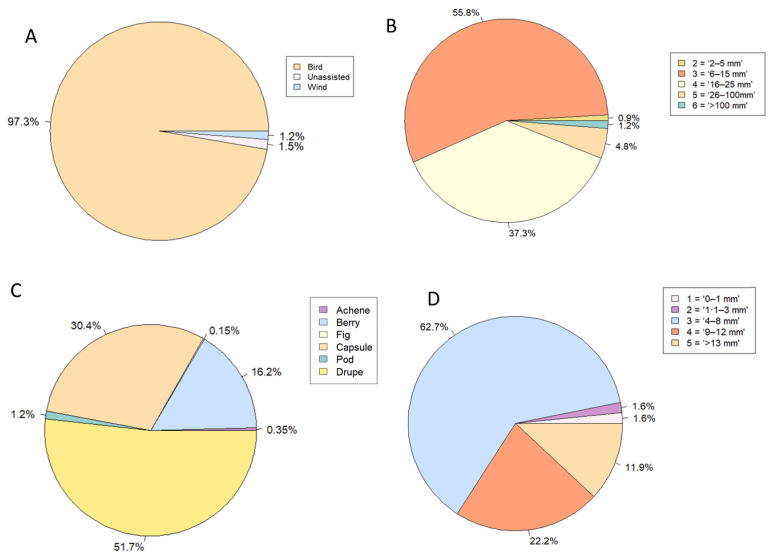
Representation of seedlings (% of the total population) for each trait state category in each plant trait. (**A**). Dispersal mechanism, (**B**). Fruit size, (**C**). Fruit type, (**D**). Seed size.

Bird dispersal was the most common dispersal mechanism category, with 97% of recorded seedlings assigned to this category (Figure 5A). The most common fruit size category recorded was category 3 (‘6–15 mm × 6–15 mm’), with 56% of seedlings producing fruit that fell into this size range (Figure 5B). No species producing fruit in size category 1 ‘<2 mm × <2 mm’ was recorded. Drupe, a fleshy, thin-skinned fruit with a seed in the middle, was the most common fruit type observed, with 52% of seedlings producing this type of fruit (Figure 5C). The most common seed size category noted was category 3 (‘4–8 mm × 4–8 mm’), with 63% of seedlings producing seeds that fall into this size range (Figure 5D). 

**Figure A3:**
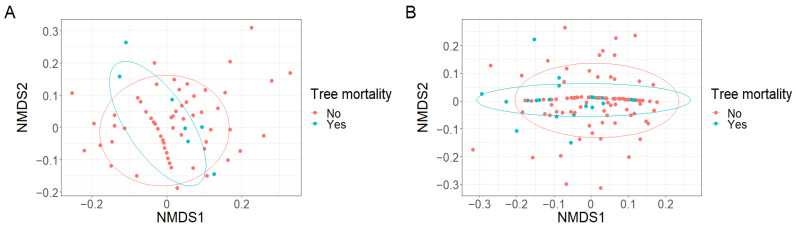
Some grouping of plots based on (**A**) fruit size trait state richness (k = 2, stress = 0.11) and (**B**) fruit size trait state richness adjusted for seedling abundance (k = 2,s stress = 0.10) was observed in response to tree mortality.

**Figure A4:**
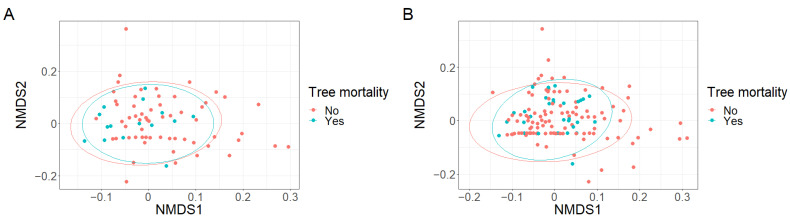
No grouping of plots based on (**A**) the richness of fruit type categories (k = 2, stress = 0.11) or (**B**) the richness adjusted for seedling abundance of fruit type categories (k = 2, stress = 0.10) was observed in response to tree mortality.

**Figure A5:**
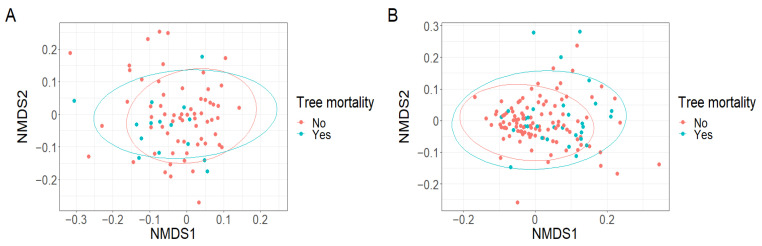
No grouping of plots based on (**A**) the richness of seed size categories (k = 2, stress = 0.15) and (**B**) the richness of seed size categories adjusted for seedling abundance (k = 2, stress = 0.11) was observed in response to tree mortality.

In all PerMANOVA tests, including those conducted on fruit size data, tree mortality explained a very small amount of the total variation (r^2^ ≤ 0.03; Appendix C, Table A1). Tree mortality did not appear to have a marked effect on the richness of categories or the richness of categories weighted for seedling abundance for fruit type, seed size, or dispersal mechanism using nMDS plots (Appendix E and Appendix F, Figure A4 and Figure A5) or PerMANOVA analyses (Appendix C, Table A1). There was some limited grouping of plots based on the richness of fruit size categories and fruit size categories weighted for seedling abundance in response to tree mortality observed in the nMDS plots (Appendix D, Figure A3). This was confirmed by a significant effect detected using a PerMANOVA analysis (F = 4.22, *p* < 0.01; F = 4.95, *p* < 0.01; Appendix C, Table A1). Indicator species analysis suggested that there was no significant driver of the grouping of plots, only reflecting a weak association and size category 6 (>100 mm) for the no tree mortality group using trait state richness (InVal = 0.33, *p* = 0.05) and trait state richness weighed by seedling abundance (InVal = 0.34, *p* = 0.05) datasets. 

## 3. Discussion

While myrtle rust has changed the species composition of the study site by killing susceptible Myrtaceae, this study has identified that species richness in the seedling layer does not appear to be affected, and most flow-on effects are likely to be temporary, particularly in relation to birds foraging. However, some questions remain over the consequences of losing some of the Myrtaceae, the ecological role species like *Archirhodomyrtus beckleri* play as an early colonizer species for rainforest regeneration, and if other species, Myrtaceae or non-Myrtaceae, will replace this species in these ecosystems. Understanding these factors and their influence on wet sclerophyll ecosystems, in general, will contribute to a better understanding of the impacts of myrtle rust.

An important finding of this study is that the loss of key Myrtaceae species is unlikely in the immediate future to cause a shift in the dominant seed dispersal mechanism at the study site. Bird dispersal was the most common seed dispersal mechanism (>90%) for tree seedlings observed in this study. The five Myrtaceae species (*Acmena smithii, Archirhodomyrtus beckleri*, *Decaspermum humile*, *Gossia hillii*, and *Rhodamnia maideniana*) observed by Pegg et al. [7] are all bird dispersed and were estimated to comprise ~90% of the midstory and ~60% of the understorey at the beginning of monitoring at the site in 2014. This is unsurprising as over two-thirds of southeast Queensland rainforest plants are likely animal dispersed [25], and all native tree species surveyed in this study in the mid- and understory of this wet sclerophyll forest are classified as rainforest species [26]. However, myrtle rust is likely to reduce the availability of fruit for birds in the short term. Prior to the arrival of myrtle rust, dominant species, such as *Gossia hillii*, would produce large fruit crops to which birds were attracted (Cook, D. 2022, personal communication). It will take several years for many of the seedlings surveyed in this study to reach maturity, and until this time, it is logical to assume there is a significant reduction in fruit availability. For example, *Decaspermum humile* is a key food source for bower bird species in other areas of southeast Queensland during the winter and early spring periods when fruit availability is lowest [27]. Species loss at the study site and others where this species is common may then affect the foraging behavior of bower birds. Also, the rose-crowned fruit dove (*Ptilinopus regina*) is known to consume fruits of *Archirhodomyrtus beckleri*, *Decaspermum humile,* and *Rhodamnia rubescens* [27,28,29] and has been observed at the study site. However, the absence of baseline bird visitation data and the scarcity of data on the identity of dispersers for some Myrtaceae species, such as *Rhodamnia maideniana*, make it difficult to draw strong conclusions on short- and long-term impacts. Further research is recommended to determine how frugivore visitation patterns may be affected by myrtle rust.

Based on the fruit size of these species recorded in the seedling layer, the loss of the Myrtaceae species due to myrtle rust is unlikely to cause a shift in bird browsing patterns. The most common fruit size category recorded in the seedling community was size category 3, ‘6–15 mm × 6–15 mm’. Four of the five key Myrtaceae species (*Archirhodomyrtus beckleri*, *Decaspermum humile*, *Gossia hillii,* and *Rhodamnia maideniana*) produce fruit falling into this same size category. This is important as gape size, the size to which a bird’s beak can open, is known to strongly correlate with fruit size, providing insight into fruit preference [21,30]. The results of this study suggest that the eventual replacement of dead and dying Myrtaceae by species recorded in the seedling layer is unlikely to cause a marked change in fruit size and, by extension, likely to have a limited effect on browsing preference based on this attribute.

The loss of key Myrtaceae species may cause a shift towards plant species that produce drupes rather than berries, which may affect browsing preferences. While drupes were the most common fruit type in the seedling community, the five key Myrtaceae species produce berries. Drupes tend to contain one or two larger seeds, while berries tend to contain many smaller seeds [25]. This trade-off between seed size and seed number is well established in ecology [31]. There is also some evidence to support a trade-off in investment between seed size and reward for dispersers in terms of the amount and quality of fruit pulp [32,33,34]. A review of frugivore species across the tropics and some subtropical regions, including Australia, suggested that specialized frugivores, such as fruit doves, favor fruits with larger seeds and a higher nutrient content, while generalist species target smaller fruits that contain a larger number of seeds with a lower nutrient value, such as berries [34]. For example, species within the Lauraceae family tend to favor the former strategy, producing drupes with a high lipid content [34]. Species from this family provide a key food source for specialist frugivores at a global level and within the tropical and subtropical forests of Australia [21,34,35]. Seedlings from two species in the Lauraceae family, *Neolitsea dealbata* (R.Br.) Merr. and *Cryptocarya glaucescens* R.Br., comprised over 40% of the total seedlings in this study. If this trajectory continues, it may benefit some true frugivores that consume their fruit, such as the topknot pigeon (*Lopholaimus antarcticus*) [25]. Further monitoring is recommended to determine if these patterns persist as the seedlings mature and if there is a corresponding shift in bird visitation.

Fruits that contained seeds falling into size category 3 (“4–8 mm × 4–8 mm”) were the most common in the seedling community at the study site. In contrast, only one of the five key Myrtaceae species (*Gossia hillii*) produced seeds falling into this seed size category, while three species (*Archirhodomyrtus beckleri*, *Decaspermum humile,* and *Rhodamnia maideniana*) were allocated to lower seed size categories (2 and 1). The loss of these Myrtaceae species could indicate a shift towards plant species that produce fruits that contain larger seeds. There tends to be a positive association between seed size and shade tolerance [36], suggesting that the degree to which disturbance affects canopy openness is an important filter for this plant trait. The secondary forest community at the study site appears to reflect two significant disturbance events. Land clearance for grazing in the late 1800s and the recent outbreak of myrtle rust. It is likely that the small-seeded early colonizer species from seed size category 1, such as *Archirhodomyrtus beckleri*, *Rhodamnia rubescens*, and *Rhodomyrtus psidioides*, that were dominant in the mid- and understorey of the study site were recruited as seedlings at a time when the overstory of Eucalypts and *Lophostemon confertus* (R.Br.) Peter G. Wilson & J.T. Waterh. was still establishing. In contrast, the seedling community that was recruited after the arrival of myrtle rust, dominated by larger-seeded, later-colonizer species in seed size category 3, such as *Neolitsea dealbata*, established itself in a community where the overstory remained intact. In this regard, any changes in light availability are a result of changes in the density of the midstory and understory only.

In areas of secondary forests across the valley and on the plateau above, these Myrtaceae species seemed to have established dense stands, appearing to form a frontier expanding into open areas (further research is needed to study these observations). The loss of these Myrtaceae species could mean the loss of species that were early colonizers. There is limited information on the autecology of other co-dominant Myrtaceae species, *Gossia hillii*, *Decaspermum humile,* and *Rhodamnia maideniana*, in these environments, making their roles difficult to articulate. However, their moderate density in some areas of the valley suggests that they may play a similar role in this system. Further research is needed to determine if other early colonizer species present in the seedling community, such as *Glochidion ferdinandi* [37], may fill this role. Further research is also recommended to determine if other secondary forests in early phases of recovery are similarly at high risk of myrtle rust infection and whether this might be the characteristic used to prioritize sites for myrtle rust management.

Wet sclerophyll forest dynamics are complex and not well studied, particularly in southeast Queensland [38]. Myrtle rust impacts on species survival and frequency may affect the structure and trajectory of this type of forest community in the short term. The defining characteristic of wet sclerophyll forests is a canopy of *Eucalyptus* sp. or functionally similar Myrtaceae genera, such as *Lophostemon* sp. [39]. The regeneration of many of these canopy species requires disturbance, often fire, to increase light availability and create a seed bed conducive to seedling germination [40]. No seedlings of *Eucalyptus* sp. or *Lophostemon confertus* were observed at the study site, suggesting that any change in canopy openness created by myrtle rust impacts was not sufficient to facilitate their regeneration.

A few seedlings from the key Myrtaceae species were observed, highlighting the impact that myrtle rust has had on their populations and regeneration potential. Seedlings from *Archirhodomyrtus beckleri*, *Decaspermum humile*, *Gossia hillii*, and *Rhodamnia maideniana* were recorded individually, representing <1% of the total seedlings surveyed. Populations of these species have experienced significant declines at the study site since the establishment of myrtle rust, with as high as 80% mortality for species like *Decaspermum humile* [7,41]. Further seedlings of two of these species, *Gossia hillii*, and *Rhodamnia maideniana*, were not observed in a recent Queensland-wide survey [42], making the presence of any seedlings unexpected. It is impossible to determine whether the seedlings recorded in this study germinated in response to the recent rainfall events or had already been recruited prior to the arrival of myrtle rust. Most seedlings exhibited symptoms of myrtle rust infection (data not included). Further monitoring is recommended to determine if these seedlings can persist in the long term.

Contrary to expectations, neither plant species richness in the understory nor canopy openness (LAI in this study) were observed to correlate with disease impact level. This contrasts with a recent study by Fernandez-Winzer [16], who observed lower species richness and seedling abundance in plots with *Rhodamnia rubescens* canopies infected by myrtle rust when compared to those with healthy canopies. Further, this study observed an increase in canopy transparency over time in the canopies of infected trees. This second result suggests a likely increase in canopy openness below trees with higher infection levels. This suggestion is supported by the results of an earlier study conducted at the study site, where a negative relationship between tree mortality and canopy cover (%) was observed [41]. A considerable difference in annual rainfall may be a key factor driving this difference in results. Both previous studies were conducted over some very low rainfall years, with 2019 being one of the driest on record in many parts of eastern Australia. For example, an estimated rainfall of 772 mm was recorded in the study region in 2019. In contrast, high rainfall was recorded in the following two years, when the study took place, with 2304 mm recorded in the region in 2020 [43]. The germination of some rainforest species and many plant species, in general, is highly responsive to soil moisture [44]. It is possible that the high rainfall after such a dry period induced a flush of germination in the seed bank. Further, increases in soil moisture can induce the production of new leaves on some mature tree species [45]. It is likely that the recent high rainfall and reduced competition for resources facilitated the growth of the canopies of eucalypts and remaining rainforest trees into the gaps created by myrtle rust tree mortality. An increase in soil nutrient levels may also have affected canopy growth. Tree mortality induced by other plant pathogens has been observed to lead to increases in soil nutrient levels [46]. However, the potential effects of myrtle rust on soil nutrients have not been investigated and are not recommended.

Seedling species diversity was highly variable between the plots sampled. Plots that had lower species richness and Shannon’s diversity index scores were characterized by a single species, *Acmena smithii*. While plots that had higher scores were characterized by nine species (i.e., *Syzygium oleosum*, *Glochidion ferdinandi*, *Elaeocarpus obovatus*, *Cryptocarya microneura*, *Acacia disparrima*, *Gossia hillii*, *Toona ciliata*, *Wilkiea huegeliana,* and *Cryptocarya laevigata*). A key aspect of successful seedling recruitment is the availability of space and resources [47]. *Acmena smithii* is one of the Myrtaceae identified by Pegg et al. [7] as being previously dominant at the site. This species is relatively tolerant to myrtle rust, so it did not experience the same level of population decline as other Myrtaceae species at the site [7], and it represented 10% of the total seedlings recorded in the current study. Most of the seedlings of this species were observed to be larger, with saplings from this species also quite common (data not included), suggesting they were likely established before the recent germination event. It is possible that areas, where *Acmena smithii* saplings were located, represented less favorable locations for seedling germination, despite increased rainfall, and were established due to a reduction in the availability of other resources such as light or nutrients.

Tree mortality appeared to have a limited correlation with the richness of fruit size categories. There was a very weak effect of tree mortality on the richness of fruit size categories; however, it was not observed to affect the richness of any other traits. Environmental factors, such as light, can act as a filter, selecting species with trait states that are most advantageous in those conditions. The removal or weakening of one of these filters can result in changes in trait state richness [22]. The absence of an effect of myrtle rust on the richness of most traits could indicate that the disturbance brought about by myrtle rust was not sufficient to significantly change these overarching community assembly filters. However, it may also be that the pathogen has not been established in the community for long enough to see a definitive change. Significant impacts of myrtle rust were only detected at the study site relatively recently, in 2014 [5]. For example, Lovett et al. [48] observed a strong negative relationship between soil carbon to nitrogen ratio and stage of disease progression using a chronosequence in a forest community in a region infected by beech bark disease as early as the 1950s. Further monitoring at the study site is recommended to determine if the current pattern continues as the disease outbreak progresses.

## 4. Materials and Methods

**Figure 6:**
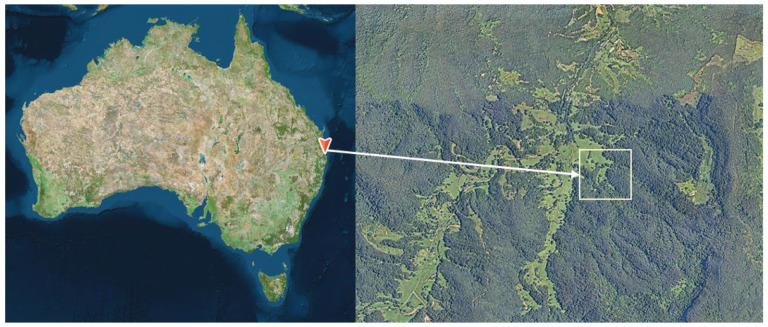
Map of the study site in the Tallebudgera Valley, southeast Queensland, created in Nearmap [50].

The study was conducted in the Tallebudgera Valley in the Gold Coast Hinterland in southeast Queensland (−28.20115, 153.35052). In a tall open forest with vine forest understorey or wet sclerophyll forest (Regional Ecosystem 12.11.2) under the Queensland Regional Ecosystem classification [49]. Notable canopy species include *Eucalyptus grandis* W.Hill and *Lophostemon confertus*, growing over a diverse rainforest understorey. This ecosystem is estimated to cover 14,000 ha of Queensland [49]. Much of the forest at the site is regrowth after past land clearing for agriculture in the 1890s and is still bordered by pastoral land (Figure 6). The average rainfall for the region is 1587 ± 457 mm recorded at a station 11 km from the study site [43]. There has been considerable variation in rainfall in the last five years, with the second driest year on record, at 772 mm, recorded in 2019, followed by several above-average rainfall years, including 2020, at 2304 mm, which was the seventh wettest year since records began in 1899 [43]. Myrtle rust impacts were first observed in native vegetation at the site in 2014 as part of a wider survey [5]. The forest at this site and in the surrounding valleys are well suited to research investigating community-level impacts of myrtle rust as they contain a high richness and abundance of highly susceptible Myrtaceae species. Tree mortality has been monitored in plots at the site between 2014 and 2020 [7,41]. High levels of tree death were recorded during this period, with 44% mortality observed in the midstory layer of the forest [7,41].

**Figure 7:**
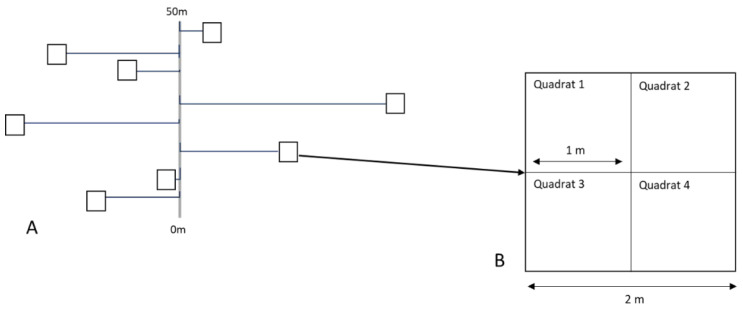
Diagram highlighting an example of the random placement of plots with reference to a line transect (**A**) and plots comprising four quadrats (**B**).

The four 50 m line transects established by Pegg et al. [7] and two additional randomly placed 50 m line transects were used as a base to determine the location of the forty-eight plots used in this study. Random allocation was used to determine the direction and distance of the plots from the transect line [51]. Plots comprised four 1 m^2^ quadrats, within which all assessments were conducted (Figure 7). 

The disease impact level was assessed using several approaches. Due to the small assessment area in this study, few quadrats contained more than one tree, and as a result, disease impact level was assessed as the presence or absence of dead trees. In this study, trees that exhibited basal sprouting were counted as dead as the canopies were dead with no leaves remaining; this represented 8 of the 68 dead trees recorded. It is acknowledged that it is not possible to know definitively that all recorded dead trees were killed by myrtle rust; however, the results of the long-term monitoring at the site suggest this has been the main cause of tree mortality since 2014, with no other significant pathogen or insect outbreaks recorded. A positive relationship between disease impact level and canopy cover was previously observed at the site [41], suggesting that measures of canopy openness can be used as proxies for disease impact level. Leaf area index (LAI) was used as an assessment of canopy openness in this study, measured using a LICOR LAI-2200C Plant Canopy Analyzer.

**Figure A6:**
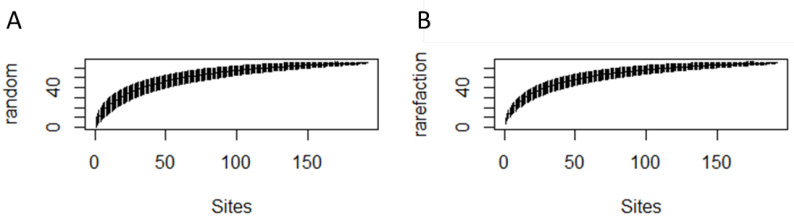
Species accumulation curves for the 192 quadrats surveyed at the site, calculated using the “exact” function (**A**) and the “rarefaction” function (**B**).

Botanical surveys were conducted in the 192 quadrats across the site. Species accumulation curves were used to determine if sampling was sufficient using the Specaccum package in Vegan (Appendix G, Figure A6). All tree seedlings (≥1 m) were identified to the species level using keys [52,53,54] and expert advice from qualified botanists (McDonald W.J.F. 2021, personal communication). This data was used to calculate Shannon’s richness index and species richness in each quadrat.

Key reproductive and dispersal traits associated with cross-trophic-level interactions were identified from the literature (seed size, fruit size, fruit type, and dispersal mechanism) [55,56,57]. Seed size categories and fruit size categories were extracted from the same sources (fruit size dimensions of 1 = ‘<2 mm × <2 mm’, 2 = ‘2–5 mm × 2–5 mm’, 3 = ‘6–15 mm × 6–15 mm’, 4 = ‘16–25 mm × 16–25 mm’, 5 = ‘26–100 mm × 26–100 mm’, 6 = ‘>100 mm in any dimension’, and seed size dimensions of 1 = ‘0–1 mm × 0–1 mm’, 2 = ‘1·1–3 mm × 1·1–3 mm’, 3 = ‘4–8 mm × 4–8 mm’, 4 = ‘9–12 mm × 9–12 mm’, 5 = ‘>13 mm in any dimension’). Trait values for each species were determined in a literature review [25,26,52,58,59,60,61,62,63,64,65], and in some cases, expert advice was needed to determine dispersal mechanisms (Catterall, C.P. 2022, personal communication) (Appendix H, Table A2). Where size ranges were quoted, the largest value was taken, and where there was variation between sources, the median was used.

The data was analyzed using R Studio software [66]. Linear mixed effects models using the lme4 package [67], with a plot set as a random effect, were used to investigate relationships between LAI or tree mortality, and species richness or Shannon’s diversity index. Species richness data and LAI data were square root transformed to satisfy assumptions of normality.

**Figure A7:**
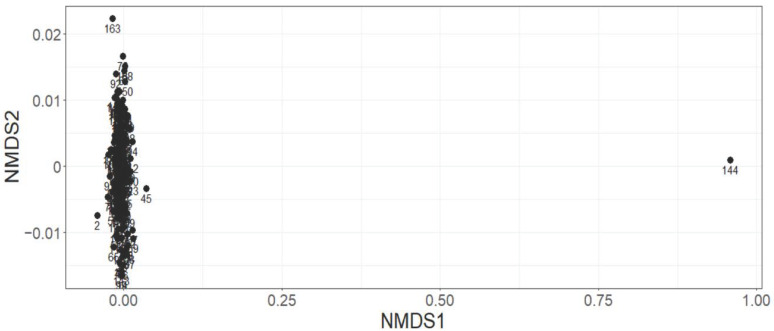
Plot 144 is shown as a significant outlier. A closer inspection of the composition of this plot showed that just two seedlings, one *Eupomatia laurina* and one *Pilidiostigma glabrum*. These species were recorded at low abundance, just three seedlings of *Eupomatia laurina* and two of *Pilidiostigma glabrum*, and never observed together in any other plot suggested to create the high dissimilarity observed. No significant effect of tree mortality was observed (F = 1.75, *p =* 0.12) using a PerMANOVA test.

Species composition data were transformed prior to running an ordination analysis. A Hellinger transformation [68] was applied, followed by a Bray Curtis distribution, using the decostand and vegdist functions in the vegan package [69]. Three of the quadrats did not contain seedlings, and as it is not possible to conduct a Bray-Curtis distribution on empty cells, these were removed. Non-parametric multidimensional scaling (nMDS) analyses were run using the metaMDS function in the Vegan package [69]. One significant outlying plot was observed (Appendix I, Figure A7); as nMDS plots are a visual analysis, this data point was removed to allow interpretation of the model. Subsequent PerMANOVA analyses were run with and without this plot, giving similar results (Appendix I, Figure A7). PerMANOVA analyses were run using the pairwise Adonis package [70], and tree mortality (yes or no) was set as the grouping variable. This analysis was selected as it has been found to be highly robust and can handle unbalanced designs and zero inflation [71,72,73]. The heterogeneity of dispersion between groups was tested before running the analysis using the betadisper function in the Vegan package.

Plant trait data were transformed in Excel (Version 2212) prior to analysis to represent a matrix of functional richness using methods provided by Katovai et al. [55]. A Gower distance matrix was applied in r before an nMDS analysis and PerMANOVA analysis were conducted on the datasets for each plant trait. An nMDS analysis was not possible for dispersal mechanism data as there was very low variance in trait state allocation, with >90% of seedlings falling into the bird dispersed category.

Cluster analysis was run as a complementary analysis to nMDS on species composition data. The same data transformation was applied as prior to nMDS. Analysis was run using the hclust function, with Ward’s [74] minimum variance method selected, a commonly applied method in ecology and effective when compared to other available methods [75,76]. The appropriate cluster number was determined using the Dunn test and the average silhouette using the Nbclust package [77]. Indicator species analysis [78] was run to determine which species were driving the clustering using the multipatt function from the indicspecies package [79]. Graphics were created using ggplot2 [80] and the patchwork package [81].

## 5. Conclusions

This study provides insights into the impacts of myrtle rust tree mortality on seedling communities. Myrtle rust, in combination with higher rainfall, is likely contributing to a shift in species composition away from the few previously dominant, highly susceptible Myrtaceae species towards a diversity of other tolerant and non-host species. The diversity of wet sclerophyll communities and their evolution with disturbance could indicate that they will be more resilient to the impacts of myrtle rust in the long term than other forest types dominated by Myrtaceae, such as Melaleuca forests. A comparison of key Myrtaceae species and the seedling community suggests that there may be shifts towards species that produce drupes and those that produce larger seeds. These shifts and the current reduction in fruit availability could affect the visitation patterns of local bird species. The loss of a suite of highly susceptible early colonizer species, such as *Archirhodomyrtus beckleri*, from the community may mean that this key role is no longer filled, affecting the capacity of this type of forest community to recover from significant disturbance events. Disease impact level was not observed to correlate with the richness of species or traits in the seedling community in this study during a high rainfall time period, while previous studies at the same site during drought conditions observed a decrease in species composition where myrtle rust was more prevalent. Finally, this result highlights the importance of long-term monitoring to detect the effects of varying climates in complex and long-lived ecosystems like tropical and subtropical forests.

## Data Availability

The data presented in this study are openly available in UQ eSpace at https://doi.org/10.48610/1e3afb5 (accessed on 17 March 2023), reference number [82].

## Appendix C

**Table A1:** Key test statistics from PerMANOVA analysis testing the effect of tree mortality on key plant traits. ** indicate significant results.

Plant Trait	r^2^	F Value	*p* Value
Fruit size—number of trait states	0.02	4.22	<0.01 **
Fruit size—number of trait states weighted by seedling abundance	0.03	4.95	<0.01 **
Fruit type—number of trait states	<0.01	0.53	0.87
Fruit type—number of trait states weighted by seedling abundance	<0.01	0.54	0.73
Seed size—number of trait states	0.01	1.13	0.28
Seed size—number of trait states weighted by seedling abundance	0.01	2.20	0.20
Dispersal mechanism—number of trait states	0.01	1.93	0.07
Dispersal mechanism—number of trait states weighted by seedling abundance	0.01	1.34	0.20

## Appendix H

**Table A2:** List of species observed in seedling survey and trait category allocation based on the literature [25,26,52,58,59,60,61,62,63,64,65], and expert advice (Catterall, C.P. 2022, Personal communication) as indicated by marked cells (*).

Species	Fruit Type Category	Fruit Size Category	Seed Size Category	Dispersal Mechanism
*Acacia disparrima* M.W. McDonald & Maslin	Pod	6	3	Bird
*Acmena smithii* (Poir.) Merr. & L.M. Perry	Berry	4	4	Bird
*Acronychia pubescens* C.T. White	Drupe	4	3	Bird
*Aphananthe philippinensis* Planch.	Drupe	3	3	Bird
*Archirhodomyrtus beckleri* (F. Muell.) A.J. Scott	Berry	3	1	Bird
*Ardisia crenata* Sims	Drupe	3	3	Bird
*Beilschmiedia elliptica* C.T. White	Drupe	3	4	Bird
*Canarium australasicum* (F.M. Bailey) Leenh.	Drupe	3	3	Bird
*Cinnamomum camphora* (L.) J. Presl	Drupe	3	3	Bird
*Cryptocarya foetida* R.T. Baker	Drupe	3	4	Bird
*Cryptocarya glaucescens* R.Br.	Drupe	4	5	Bird
*Cryptocarya laevigata* Blume	Drupe	5	5	Bird
*Cryptocarya microneura* Meisn.	Drupe	3	4	Bird
*Cryptocarya obovata* R.Br.	Drupe	3	4	Bird
*Cryptocarya triplinervis* R.Br.	Drupe	3	4	Bird
*Cupaniopsis newmanii* S.T. Reynolds	Capsule	4	5	Bird
*Cyclophyllum coprosmoides* (F. Muell.) S.T.Reynolds & R.J.F. Hend.	Drupe	4	3	Bird
*Daphnandra tenuipes* Perkins	Achene	3	2	Wind
*Decaspermum humile* (G.Don) A.J. Scott	Berry	3	2	Bird
*Denhamia celastroides* (F.Muell.) Jessup	Capsule	4	2	Bird *
*Diploglottis australis* Radlk.	Capsule	4	4	Bird
*Elaeocarpus grandis* F. Muell.	Drupe	5	4	Bird
*Elaeocarpus Kirtonii* F.Muell. ex F.M. Bailey	Drupe	3	3	Bird
*Elaeocarpus obovatus* G. Don	Drupe	3	3	Bird
*Endiandra discolor* Benth.	Drupe	4	5	Bird
*Endiandra globosa* Maiden & Betche	Drupe	5	5	Ground vertebrates (mainly now extinct bird/mammal megafauna) *
*Eupomatia laurina* Hook.	Achene	5	3	Bird
*Euroschinus falcatus* Hook.f.	Drupe	3	3	Bird
*Ficus coronata* Reinw. ex Blume	Fig	5	1	Bird
*Flindersia bennettii* F.Muell. ex C. Moore	Capsule	5	3	Wind
*Glochidion ferdinandi* (Müll.Arg.) F.M. Bailey	Capsule	4	3	Bird
*Gossia hillii* (Benth.) N.Snow & Guymer	Berry	3	3	Bird
*Guioa semiglauca* (F.Muell.) Radlk.	Capsule	3	3	Bird
*Hedraianthera porphyropetala* F. Muell.	Capsule	5	4	Bird *
*Homalanthus populifolius* Graham	Capsule	3	3	Bird
*Hymenosporum flavum* F. Muell.	Capsule	5	4	Wind
*Jagera pseudorhus* (A.Rich.) Radlk.	Capsule	4	3	Bird
*Karrabina benthamiana* (F.Muell.) Rozefelds & H.C. Hopkins	Capsule	4	3	Wind
*Lantana camara* L.	Drupe	2	3	Bird
*Litsea reticulata* Benth. & Hook.f. ex F. Muell.	Drupe	3	4	Bird
*Mallotus philippensis* (Lam.) Müll.Arg.	Capsule	3	2	Bird
*Mischocarpus pyriformis* (F.Muell.) Radlk.	Capsule	4	4	Bird
*Myrsine variabilis* R.Br.	Drupe	3	3	Bird
*Neolitsea dealbata* (R.Br.) Merr.	Drupe	3	3	Bird
*Notelaea longifolia* Vent.	Drupe	4	4	Bird
*Ochna serrulata* Walp.	Drupe	3	3	Bird
*Olea paniculata* R.Br.	Drupe	3	4	Bird
*Pilidiostigma glabrum* Burret	Berry	3	3	Bird
*Pittosporum revolutum* Dryand.	Capsule	5	3	Bird *
*Pleioluma queenslandica* (P. Royen) Swenson	Drupe	4	5	Bird
*Psychotria loniceroides* Sieber ex DC.	Drupe	3	3	Bird
*Psydrax lamprophylla* f. latissima S.T. Reynolds & R.J.F. Hend.	Drupe	3	3	Bird *
*Quintinia verdonii* F. Muell.	Capsule	2	1	Unassisted
*Rhodamnia maideniana* C.T. White	Berry	3	2	Bird *
*Sarcopteryx stipata* (F.Muell.) Radlk.	Capsule	4	3	Bird
*Senna pendula var. glabrata* (Vogel) H.S.Irwin & Barneby	Pod	6	3	Unassisted
*Synoum glandulosum* A.Juss.	Capsule	4	3	Bird
*Syzygium hodgkinsoniae* (F. Muell.) L.A.S.Johnson	Berry	5	5	Bird
*Syzygium luehmannii* (F. Muell.) L.A.S.Johnson	Berry	3	3	Bird
*Syzygium moorei* (F.Muell.) L.A.S. Johnson	Berry	5	5	Bird
*Syzygium oleosum* (F.Muell.) B. Hyland	Berry	4	4	Bird
*Tasmannia insipida* R.Br. ex DC.	Berry	4	2	Bird *
*Toona ciliata* M.Roem.	Capsule	4	5	Wind
*Trochocarpa laurina* R.Br.	Drupe	3	1	Bird
*Wilkiea huegeliana* (Tul.) A.DC.	Drupe	3	3	Bird
